# Successful annuloplasty using the cone-beam computed tomography-assisted radiofrequency thermocoagulation system in a patient with severe vertebral deformity: a case report

**DOI:** 10.1186/s40981-022-00554-z

**Published:** 2022-08-13

**Authors:** Shintaro Hagihara, Masayuki Nakagawa, Kana Matsubara, Kohei Godai, Kenya Kamijima, Yoichiro Abe

**Affiliations:** 1 Department of Anesthesiology and Pain Medicine, Kagoshima University Hospital, 8-35-1 Sakuragaoka, Kagoshima, 890-8520 Japan; 2 Department of Pain Clinic, NTT Medical Center Tokyo, 5-9-22 Higashigotanda, Shinagawa, Tokyo, 141-8625 Japan

**Keywords:** Radiofrequency thermocoagulation, Cone-beam computed tomography, Annuloplasty, Degenerative disc disease, Spina bifida occulta, Lumbosacral transitional vertebra

## Abstract

**Background:**

Complex anatomical features are challenging for minimally invasive intradiscal therapy owing to insufficient visualization for accurate needle advancement. We report the case of a patient with dysraphic vertebral pathologies who presented with L5/S1 degeneration and was successfully treated with annuloplasty using the cone-beam computed tomography (CBCT)-assisted radiofrequency thermocoagulation system.

**Case presentation:**

A 34-year-old woman presented with a lower back and left radicular pain of L5/S1 discogenic origin, accompanied by spina bifida occulta and lumbosacral transitional vertebra. Radiofrequency annuloplasty was performed to preserve disc height and spinal stability, with real-time CBCT guidance for the congenital and degenerative conditions. The procedure relieved her left lower-extremity pain and magnetic resonance imaging revealed that the L5/S1 disc bulging decreased while the disc height was preserved.

**Conclusion:**

Optimal accessibility of radiofrequency thermocoagulation and effective needle guidance using CBCT significantly improve the success rate of annuloplasty at the L5/S1 degenerative disc with severe vertebral deformity.

## Background

Image guidance plays a significant role in accurate needle placement for minimally invasive intradiscal therapy [1–3]. Vertebral abnormalities are a challenge for treating lumbar disc diseases using conventional C-arm fluoroscopy because anatomical landmarks are insufficiently visualized for needle advancement [1–3].

C-arm fluoroscopic cone-beam computed tomography (CBCT) is an advanced three-dimensional imaging technology available on flat panel-based angiography systems. CBCT with special needle guidance software provides high-resolution image constructions, enabling real-time image guidance during a procedure [1, 2].

**Fig. 1:**
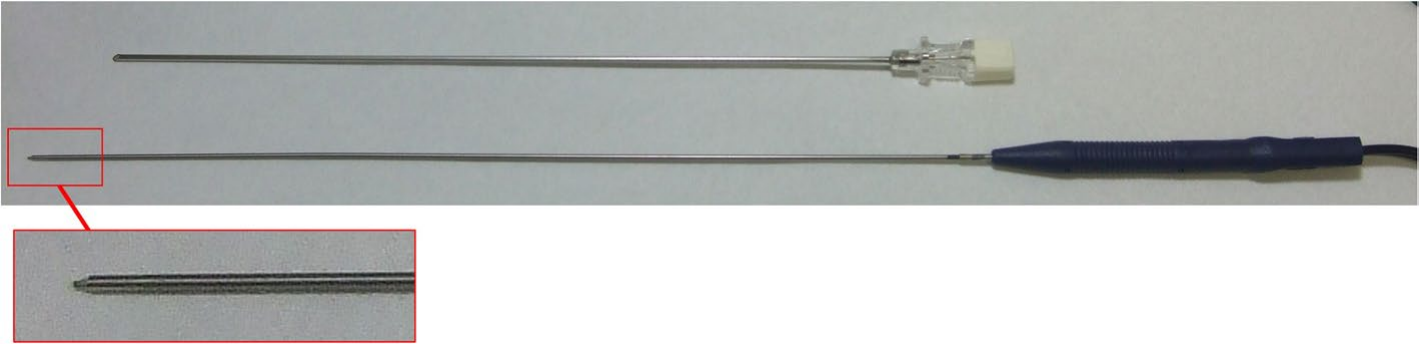
The radiofrequency thermocoagulation system. A 16-gauge spine needle is used as a cannula through which a 1-mm-diameter radiofrequency thermocoagulation probe is placed

Radiofrequency annuloplasty is a minimally invasive technique wherein radiofrequency thermal energy is delivered to the bulging part of the disc to treat lower back and leg pain [4]. A 1-mm-diameter radiofrequency thermocoagulation probe (Fig. 1) [5] can reach the posterior annular surface of the L5/S1 degenerative disc in complex anatomical pathologies, which may be difficult to access for other minimally invasive procedures [3].

Herein, we report a case of L5/S1 degenerative disc disease in a patient with spina bifida occulta (SBO) and lumbosacral transitional vertebra (LSTV), wherein CBCT played a significant role in real-time needle guidance, and the radiofrequency thermocoagulation system provided optimal accessibility for annuloplasty.

## Case presentation

**Fig. 2:**
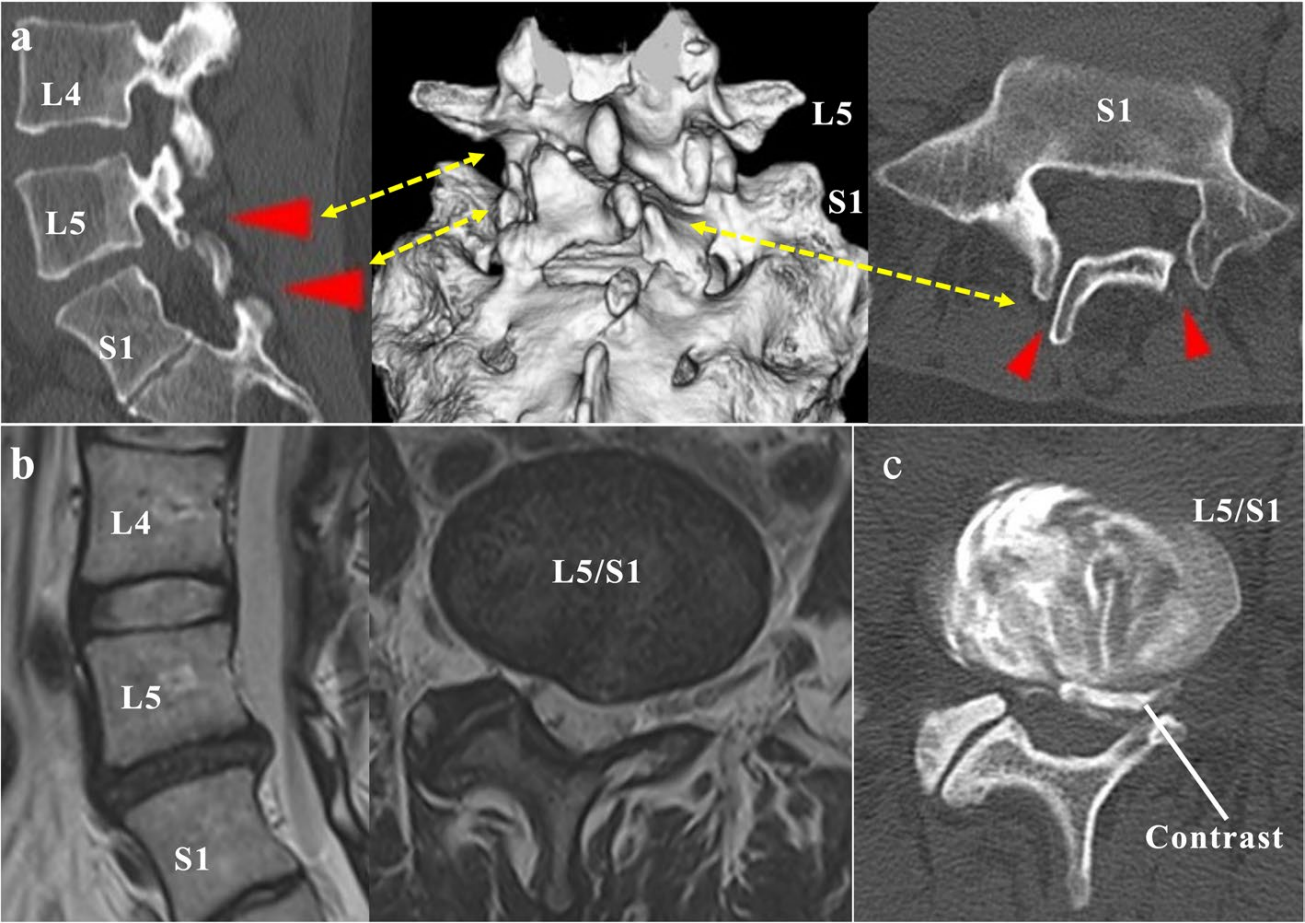
Preoperative image evaluation. **a** Sagittal, axial, and reconstructed 3-dimensional computed tomography images show the left L5/S1 abnormal facet complex and unco-ossified S1 lamina (red arrows). **b** Sagittal and axial T2-weighted magnetic resonance imaging show the L5/S1 degenerative disc compressing the existing left S1 nerve. **c** Axial computed tomography discography shows the morphologically violated disc at L5/S1. The contrast medium enters the posterior annulus

Written informed consent was obtained from the patient to publish this case report and accompanying images. After conservative treatment for three months, a 34-year-old woman (height, 159 cm; weight, 65.2 kg) presented with symptoms of left lumbar radiculopathy. She had no remarkable medical history. Her lower back and leg pain scored 8 on the numerical rating scale, radiating in the left S1 region, with a sensory deficit of 9 out of 10 and no motor weakness. The straight leg raising test was positive at 30°. Lumbar spine radiographs showed unco-ossified S1 lamina with lumbarization, narrowed L5/S1 disc space, and neuralgic scoliosis. Computed tomography (CT) demonstrated congenital S1 dysraphism and dysmorphic left L5/S1 facet complexes related to SBO (Fig. 2a). T2-weighted magnetic resonance imaging (MRI) revealed a collapsed L5/S1 disc space with a left posterior focal annular bulge (Fig. 2b). The signal intensity was intermediate to hypointense to the cerebrospinal fluid and classified as grade 4 based on Pfirrmann’s classification system [6]. The left S1 nerve root block temporarily relieved lumbar radiculopathy. L5/S1 discography revealed concordant pain. CT discography confirmed that the nucleus pulposus and posterior annulus fibrosus were poorly marginated (Fig. 2c).

To preserve disc height and spinal stability, we performed percutaneous annuloplasty. However, a technical difficulty was predicted in identifying the appropriate entry and target points using conventional C-arm fluoroscopy alone for the dysraphic vertebral and degenerative disc-related pathologies. CBCT (ARTIS Pheno; SIEMENS Healthineers, Erlangen, Germany) was chosen for needle guidance and a steerable radiofrequency thermocoagulation probe for optimal accessibility.

**Fig. 3:**
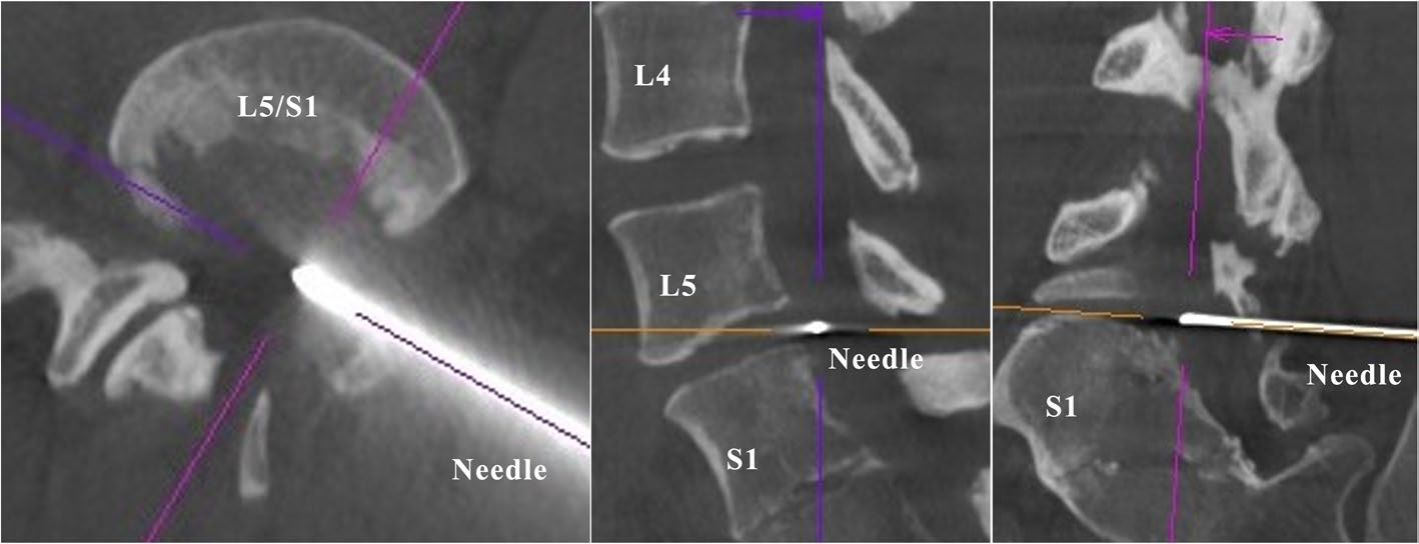
Multiplanar reconstructions for needle guidance. The real-time needle trajectory is confirmed in the axial, sagittal, and coronal oblique planes

**Fig. 4:**
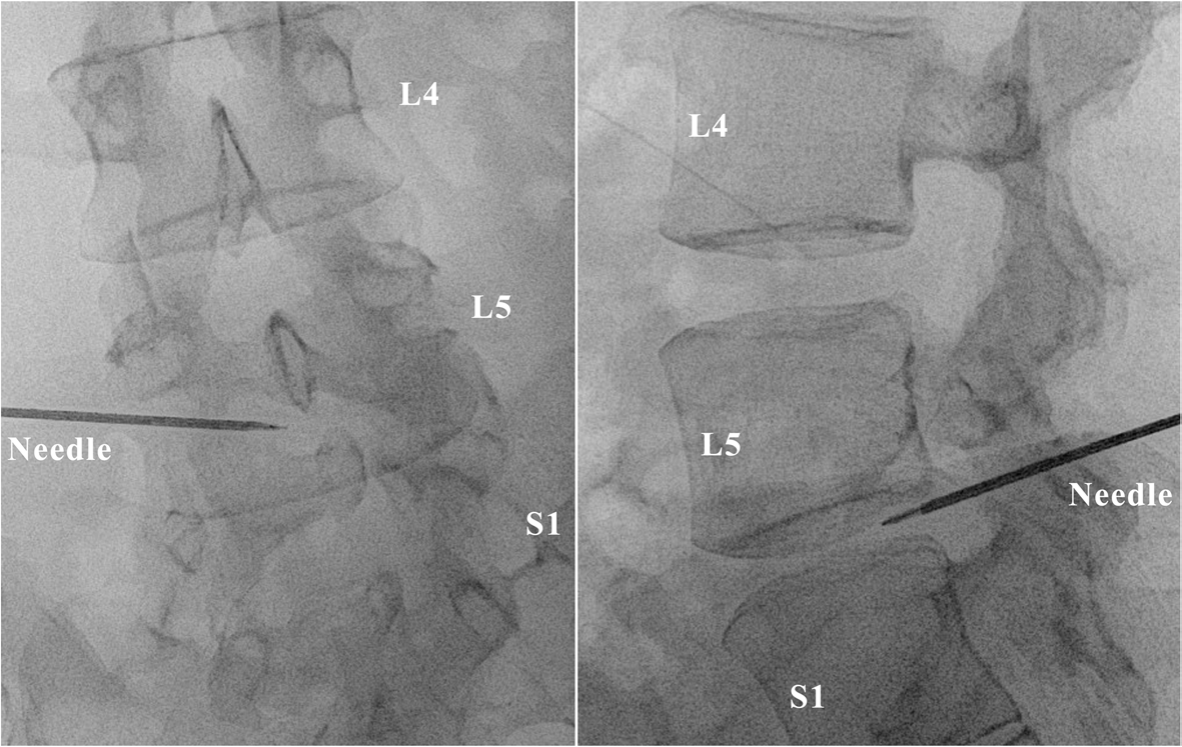
Live fluoroscopy for needle deployment. The tip of the needle is confirmed at the anteroposterior and lateral projections

The procedure was performed under light sedation using intramuscular injection of midazolam 2 mg. The patient was placed in the right lateral decubitus position for the posterolateral approach. Two board-certified interventional radiologists performed the rotational acquisition of CBCT images. Multiplanar reconstructed images were constructed to plan the desired skin entry and target points. The special navigation software automatically computed the C-arm angulations, which were positioned to the planned needle path, where the entry and target points were directly superimposed. The entry point was visible as a small circle on the fluoroscopic images, and a 16-gauge spine needle was inserted under the guidance of the planned needle path. Once the needle was advanced to the L5/S1 intervertebral disc, a second CBCT scan was performed. The positional correlation between the actual needle tip and desired target point was revised (Fig. 3). The needle was advanced for minor adjustments under anteroposterior and lateral fluoroscopic projections (Fig. 4). The stylet was removed, and the probe was placed into the needle. The radiofrequency thermocoagulation procedure was performed by Bipolar-Turbo using the Surgi-Max (Elliquence, LLC, Baldwin, NY). No intraoperative complications occurred during any of the procedures.

After 1 week from the procedure, the numerical rating scale decreased to 1 from 8 and the straight leg raising test improved to 80° from 30° and maintained at the same score for the next 1 year. Postoperative image evaluation of MRI finding at the 2 months after the treatment showed that L5/S1 disc bulging decreased; in contrast, the disc height [7] was preserved at 5.32 mm compared to that at 6.29 mm, preoperatively.

## Discussion

This case report provides two important suggestions. First, real-time image guidance of CBCT has led to technical success in needle placement. Second, the steerable radiofrequency thermocoagulation probe of Trigger-Flex Dart (Elliquence, LLC, Baldwin, NY) provided optimal accessibility to the posterior annulus at the L5/S1 degenerative disc with severe vertebral deformity.

There are many different treatment choices for lumbar disc diseases, but the most effective strategy has not been determined yet [4]. The preferred first-line treatments are physical therapy and pharmacological management, after excluding “red flags” suggesting serious pathologies [8, 9]. The less invasive options of local anesthesia and steroids, such as epidural injections and nerve root blocks, can be considered for diagnostic and therapeutic pain relief [9]. Provocative discography is essential for diagnosing lumbar disc disease [9, 10]. With negative discography, other causes need to be ruled out. With positive discography, minimally invasive percutaneous procedures can be considered, such as coagulation of the posterior annulus, decompression of the painful disc, and chemonucleolysis [4]. Among several surgical options, minimally invasive decompression surgeries have recently become popular, providing small openings with microscopic decompression and endoscopic spinal discectomy [9].

Although radiofrequency thermocoagulation is the good therapy for lumbar disc pathologies [3], it is often difficult to advance the needle at the L5/S1 degenerative disc because of degenerative changes in the spine (osteoarthritis, calcified and hypertrophic ligaments, severe scoliosis) and other anatomical structures (high iliac crest and transverse process) [2, 3]. In these cases, to locate the needle at the posterior annular surface is even more difficult [3]. We speculate that a CBCT-assisted radiofrequency coagulation system can improve the success rate of annuloplasty at the L5/S1 degenerative disc with severe spinal deformity and may preserve disc height with resultant spine stability. The L5/S1 disc diminution was within 1 mm during the procedure. Further studies are required to confirm the clinical relevance of these imaging findings.

A potent chemonucleolytic drug with condoliase reduces intradiscal pressure on the nerve root and improves lumbar disc herniation symptoms [11]. However, it may not be reasonable given the patient’s contralateral dysraphism. It may decrease disc height, disrupting coronal balance, and precipitating foraminal stenosis and radiculopathy [12]. Total disc replacement is another motion-preserving surgery, but concerns remain regarding the future of the implant in younger patients [9, 10].

CBCT-guided percutaneous nucleoplasty is highly effective in challenging lumbar disc herniation cases, with adequate procedure time and radiation dose [2]. The high spatial and contrast resolution of multiplanar reconstruction images obtained from CBCT datasets allow precise evaluation of complex anatomical and small structures that cannot be detected with conventional fluoroscopy [1, 2]. CBCT allows effective needle guidance with an accuracy of approximately 3 mm [1]. Although CBCT images are useful for preoperative planning, registration accuracy may be affected by intraoperative patient motion [13]. A second CBCT confirmation should be considered whenever periprocedural assessment of on-table patient motion is required. Acquiring additional CBCT images can revise the correct needle deployment within a range of millimeters, potentially avoiding treatment failure and improving treatment safety [1, 2, 13, 14].

The degenerative disc releases nociceptive and growth factors, causing the ingrowth of nerve fibers and neovascularization in the annulus fibrosus [15, 16]. Degenerative disc neovascularization increases proteolytic enzyme activity, precipitating disc degeneration, and weakening supporting ligaments, leading to instability [17]. The protruding degenerative disc with chemical irritation and mechanical compression contributes to discogenic and radicular pain [18]. Radiofrequency annuloplasty can cauterize the fibrotic tissues containing free nerve endings and neovascularization in the outer annulus, gradually stiffening the collagen in the annulus and diminishing the load on the disc [3, 18–20].

SBO is caused by failure of fusion between posterior vertebral elements without affecting the spinal cord or meninges, with a prevalence of 0.6–25% [21, 22]. Although no definitive causal link has been established between congenital SBO and the development of lumbar disc herniation [23, 24], SBO is suspected to be a predisposing factor for degenerative disc disease based on the hypothesis that congenital defects may cause instability of the base of the lumbar spine, therefore leading to degenerative deformities and posterior disc herniation [23]. LSTV is a congenital spinal anomaly defined as either sacralization or lumbarization [24]. LSTV is common in the general population, with a reported prevalence of 4–30% [24]. Several reports indicate that a higher incidence of degenerative disc herniation and nerve root canal stenosis are encountered at a level above the LSTV due to increased mechanical stress and spine instability [22–24]. An association between LSTV and SBO was found in 0.02% of the healthy population [23]. Although their existence may be incidental, these developmental malformations aggravate the clinical severity of the condition [21].

## Conclusion

We conclude that the technical achievements of annuloplasty with a combination of radiofrequency thermocoagulation and CBCT can be used for the L5/S1 degenerative disc with severe spinal deformity and may preserve disc height with resultant spine stability.

## Data Availability

Not applicable.

## Abbreviations

SBO: Spina bifida occulta
LSTV: Lumbosacral transitional vertebra
CBCT: Cone-beam computed tomography
CT: Computed tomography
MRI: Magnetic resonance imaging

## References

[1] Busser WM, Braak SJ, Fütterer JJ, van Strijen MJ, Hoogeveen YL, de Lange F (2013). Cone beam CT guidance provides superior accuracy for complex needle paths compared with CT guidance. Br J Radiol.

[2] Ierardi AM, Piacentino F, Giorlando F, Magenta Biasina A, Bacuzzi A, Novario R (2016). Cone beam computed tomography and its image guidance technology during percutaneous nucleoplasty procedures at L5/S1 lumbar level. Skelet Radiol.

[3] Kumar N, Zaw AS, Kumar N, Sonawane D, Hey HWD, Kumar A (2018). Annulo-Nucleoplasty using disc-Fx in the management of degenerative lumbar disc pathology: how long can the effect last? Glob. Spine J.

[4] Ravikanth R (2020). A review of discogenic pain management by interventional techniques. J Craniovertebr Junction Spine.

[5] Trigger-Flex® Dart Bipolar System. Elliquence, LLC. 2022. https://www.elliquence.com/wp-content/uploads/2016/11/SM-032-Trigger-Flex-Dart-Brochure-Rev-A.pdf. Accessed 15 May 2022.

[6] Pfirrmann CW, Metzdorf A, Zanetti M, Hodler J, Boos N (2001). Magnetic resonance classification of lumbar intervertebral disc degeneration. Spine..

[7] Zhang F, Zhang K, Tian HJ, Wu AM, Cheng XF, Zhou TJ (2018). Correlation between lumbar intervertebral disc height and lumbar spine sagittal alignment among asymptomatic Asian young adults. J Orthop Surg Res.

[8] DePalma MG (2020). Red flags of low back pain. JAAPA..

[9] Kim HS, Wu PH, Jang IT (2020). Lumbar degenerative disease part 1: anatomy and pathophysiology of intervertebral discogenic pain and radiofrequency ablation of basivertebral and sinuvertebral nerve treatment for chronic discogenic back pain: a prospective case series and review of literature. Int J Mol Sci.

[10] Manabe H, Yamashita K, Tezuka F, Takata Y, Sakai T, Maeda T (2019). Thermal annuloplasty using percutaneous endoscopic discectomy for elite athletes with discogenic low back pain. Neurol Med Chir (Tokyo).

[11] Muramatsu D, Yamaguchi H, Minamisawa Y, Nii A (2020). Selective chemonucleolysis with condoliase in cynomolgus monkeys. Toxicol Pathol.

[12] Chiba K, Matsuyama Y, Seo T, Toyama Y (2018). Condoliase for the treatment of lumbar disc herniation: a randomized controlled trial. Spine..

[13] Tam AL, Mohamed A, Pfister M, Chinndurai P, Rohm E, Hall AF (2010). C-arm cone beam computed tomography needle path overlay for fluoroscopic guided vertebroplasty. Spine..

[14] Powell MF, DiNobile D, Reddy AS (2010). C-arm fluoroscopic cone beam CT for guidance of minimally invasive spine interventions. Pain Phys.

[15] Peng B, Wu W, Hou S, Li P, Zhang C, Yang Y (2005). The pathogenesis of discogenic low back pain. J Bone Joint Surg (Br).

[16] García-Cosamalón J, del Valle ME, Calavia MG, García-Suárez O, López-Muñiz A, Otero J (2010). Intervertebral disc, sensory nerves and neurotrophins: who is who in discogenic pain?. J Anat.

[17] Fogelholm RR, Alho AV (2001). Smoking and intervertebral disc degeneration. Med Hypotheses.

[18] Ahn Y, Lee SH (2010). Outcome predictors of percutaneous endoscopic lumbar discectomy and thermal annuloplasty for discogenic low back pain. Acta Neurochir.

[19] Fukui S (2006). Changes on MRI in lumbar disc protrusions in two patients after intradiscal electrothermal therapy. J Anesth.

[20] Park CH, Lee KK, Lee SH (2019). Efficacy of transforaminal laser annuloplasty versus intradiscal radiofrequency annuloplasty for discogenic low back pain. Korean. J Pain.

[21] Taskaynatan MA, Izci Y, Ozgul A, Hazneci B, Dursun H, Kalyon TA (2005). Clinical significance of congenital lumbosacral malformations in young male population with prolonged low back pain. Spine..

[22] Seçer M, Muradov JM, Dalgiç A (2009). Evaluation of congenital lumbosacral malformations and neurological findings in patients with low back pain. Turk Neurosurg.

[23] George P, Maria T, Panagiotis K (2013). Lumbosacral transitional vertebra associated with sacral spina bifida occulta: a case report. Acta Med (Hradec Kralove).

[24] Konin GP, Walz DM (2010). Lumbosacral transitional vertebrae: classification, imaging findings, and clinical relevance. AJNR Am J Neuroradiol.

